# Kidney Microcirculation as a Target for Innovative Therapies in AKI

**DOI:** 10.3390/jcm10184041

**Published:** 2021-09-07

**Authors:** Bülent Ergin, Sakir Akin, Can Ince

**Affiliations:** 1 Department of Intensive Care, Laboratory of Translational Intensive Care, Erasmus MC, University Medical Center Rotterdam, 3015 GD Rotterdam, The Netherlands; c.ince@erasmusmc.nl; 2 Department of Intensive Care, Haga Teaching Hospital, 2545 AA The Hague, The Netherlands; sakirakin@gmail.com

**Keywords:** acute kidney injury, microcirculation, oxygenation, innovative therapies

## Abstract

Acute kidney injury (AKI) is a serious multifactorial conditions accompanied by the loss of function and damage. The renal microcirculation plays a crucial role in maintaining the kidney’s functional and structural integrity for oxygen and nutrient supply and waste product removal. However, alterations in microcirculation and oxygenation due to renal perfusion defects, hypoxia, renal tubular, and endothelial damage can result in AKI and the loss of renal function regardless of systemic hemodynamic changes. The unique structural organization of the renal microvasculature and the presence of autoregulation make it difficult to understand the mechanisms and the occurrence of AKI following disorders such as septic, hemorrhagic, or cardiogenic shock; ischemia/reperfusion; chronic heart failure; cardiorenal syndrome; and hemodilution. In this review, we describe the organization of microcirculation, autoregulation, and pathophysiological alterations leading to AKI. We then suggest innovative therapies focused on the protection of the renal microcirculation and oxygenation to prevent AKI.

## 1. Introduction

The kidney is one of the most crucial and vulnerable organs in the human body for the maintenance of normal hemostasis. The kidney plays an important role in eliminating metabolic waste products such as urea and creatinine and maintaining body water content and electrolyte balance. By doing so, the kidney controls blood pressure (BP) by regulating sodium and water excretion and by releasing renin, as a vasoactive mediator, and erythropoietin, to counteract systemic hypoxia. The kidney is one of the most vascularized organs in the human body and receives about 20–25% of the cardiac output (renal blood flow (RBF) is equal to 1–1.2 L/min) in adults [1,2]. The functional unit of the kidney, called the nephron, consists of different structural compartments including the glomerulus, bowman capsule, proximal tubule, loop of Henle, and distal tubule. While the renal artery supplies blood to the kidney, it gives rise to a complex and dynamic microcirculatory network that provides sufficient intraglomerular pressure, peritubular capillary pressure, perfusion, and oxygenation for the kidney to sustain glomerular filtration (GFR) and function.

Acute kidney injury (AKI) is a multifactorial conditions that is associated with renal tubular and glomerular damage, renal microcirculatory disturbance, hypoxia and the loss of renal function in perioperative and intensive care settings. Despite the advances in early diagnostic and treatment methods, AKI occurs in 2–18% of all hospitalized patients [3,4,5], in 22–57% of ICU patients [6,7], and in 30% of cardiac surgery patients [8,9], which is directly associated with high morbidity and mortality. The pathophysiological mechanism of AKI is still not fully understood; however, it is well known that alterations to renal microcirculation and oxygenation play a major role in renal dysfunction, inflammation, oxidative stress, and damage [10,11].

In the following review, we summarize the main components of renal microcirculation, autoregulation, and its pathophysiological alterations during different pathological states leading to AKI. Finally, we propose innovative therapies based on the protection of renal microcirculation and oxygenation to prevent AKI.

## 2. Renal Blood Circulation

**Figure 1:**
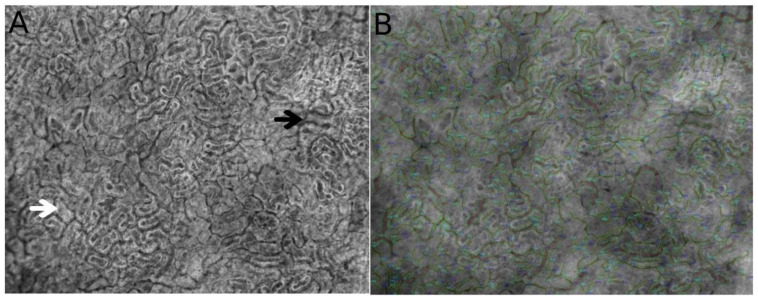
Direct observation using hand-held video microscopy (HVM) of the microcirculatory structure and cortical tubule organization in the kidney. Figures were obtained from automated microcirculatory measurement software (MicroTools) [18]. The white arrow indicates the cortical tubules, and the black arrow shows peritubular vessels around the tubules (**A**). (**B**) represents the vessel border drawn by MicroTools in the renal cortex.

The renal artery divides into interlobar and arcuate arteries and interlobular arteries before the glomerular arteriolar system. The arteriolar system in the kidney supplies blood to three microcirculatory compartments: the peritubular (Figure 1), glomerular, and outer medullary microcirculations, with blood flow being tightly regulated in the cortex and the medulla due to their different functions [12]. The renal microcirculation has unique characteristics that distinguish it from that of other tissues. Thus, an afferent arteriole (Af-Art) gives rise to the capillary structure of the glomerulus and leaves the glomerulus as an efferent arteriole (Ef-Art). The Ef-Art branches into a new group of capillaries called peritubular capillaries, which connect to the venous system via renal venules, arcuate veins, and the renal vein. Additionally, some branches of the efferent arterioles from the juxtamedullary glomerulus give rise to the descending vasa recta (DVR) and ascending vasa recta (AVR) in the outer medulla. Moreover, the DVR and (AVR) join the vascular bundle in the inner stripe of the medulla [13,14,15]. The glomerular Af-Arts are tightly regulated by regulatory systems to sustain GFR.

**Figure 2:**
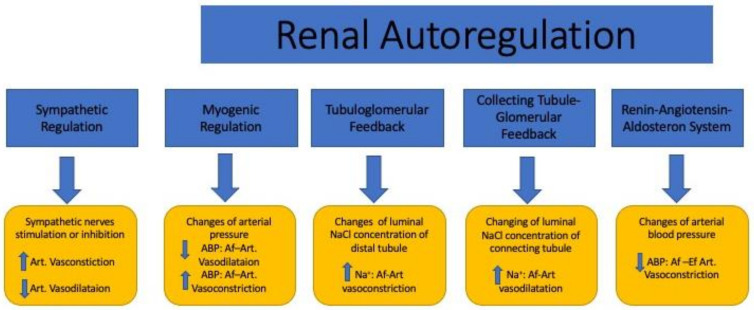
The effects of the sympathetic and renal autoregulatory systems on renal microcirculation. ABP: arterial blood pressure; Na^+^: sodium; Art: artery; Af-Art: afferent arteriole; Ef-Art: efferent arteriole; ↑: high; ↓: low.

In normal conditions, about 90% of blood plasma is filtered via active and osmotic transport between the glomerular–peritubular capillary networks and the convolute tubules in the cortical nephrons. On the other hand, in addition to glomerular–peritubular filtration, the parallel arrangement of the DVR and AVR along with the descending and ascending limbs of Henle maintain a countercurrent mechanism that promotes the cortico-medullary osmotic gradient for solute exchange and water reabsorption in the juxtamedullary nephrons. The intravascular volume and mean arterial pressure (MAP) between 80 and 180 mmHg can vary due to physiological adaptations to the environment without affecting GFR and glomerular perfusion. The regulation of blood flow and flow distribution through the cortex and medulla is achieved by regulatory systems including the paracrine (nitric oxide) system, myogenic mechanisms, tubuloglomerular feedback (TGF), connecting tubule–glomerular feedback (CTGF) [16,17], the renin–angiotensin aldosterone system (RAAS), and the sympathetic nervous system (SNS) (Figure 2).

## 3. Autoregulation of the Kidney Microcirculation

Efficient renal function requires relatively constant RBF and glomerular filtration despite fluctuations in arterial pressure. Renal vascular resistance, which is directly relevant to renal blood flow driven by arterial pressure and cardiac output, also plays a pivotal role in the distribution of blood to sustain glomerular filtration. RBF is also regionally specific and tightly regulated by autoregulation consisting of TGF, RAAS, and renal myogenic responses (Figure 2). RBF and GFR are maintained within a remarkably limited range, when renal arterial pressure is varied [19]. To maintain renal function, renal autoregulation is accomplished by different mechanisms that influence preglomerular resistance and intraglomerular capillary pressure. In the kidney, Af-Art resistance is the most efficient way to regulate glomerular perfusion, intraglomerular pressure, and GFR. The Af-Art and Ef-Art tone are regulated by complex interactions between vasodilators such as NO and prostaglandin E2 (PGE2) and vasoconstrictors such as endothelin, angiotensin II, and adenosine [19,20,21,22].

### 3.1. Myogenic Regulation

Changes in the preglomerular arteries and Af-Art vascular tone are one of the mechanisms that maintain glomerular filtration [17]. It involves the myogenic mechanical stretch of the preglomerular arteries and Af-Arts in the case of an increase in arterial pressure. The mechanoreceptors of smooth muscle cells on Af-Art walls respond to alterations in transmural pressure and rapidly adjust their diameter by vasodilatation or vasoconstriction. Moreover, the renal myogenic response in particular leads to the constriction of the preglomerular vasculature and increases transmural pressure. Thus, it is thought to play an important role in protecting the kidney from high systemic pressure-related renal injury [23].

### 3.2. Tubuloglomerular and Connecting Tubule–Glomerular Feedback

TGF is a homeostatic control mechanism designed to stabilize NaCl delivery into the late part of the nephron. Changes in luminal NaCl concentration at the macula densa (differentiated epithelial cells of the distal convolute tubule) via the Na–K–2Cl channel adjust the glomerular Af-Art vasomotor tone, which results in changes in the glomerular filtration rate [24]. The macula densa cells in the distal convoluted tubule (DCT) are sensitive to the fluctuation of amounts of filtered NaCl. A reduced sodium (Na^+^) load entering the tubule results in the inhibition of the TGF system, but it is activated by high levels of Na^+^ [25,26]. Following the activation of TGF with high Na^+^ levels, Af-Art vasoconstriction occurs by ATP/adenosine [27], which results in the reduction in GFR. The vascular tone changes are also, themselves, under the influence of nitric oxide, adenosine triphosphate (ATP), and prostaglandin E2 [12].

CTGF has recently been suggested as a novel mechanism for renal microcirculatory regulation that involves sodium handling in the connecting tubules (CNT) [28]. It has been shown that a high sodium content in CNT could cause Af-Art vasodilation initiated by activation of epithelial Na^+^ channel in a distal nephrons [16,29]. The release of prostaglandins and epoxyeicosatrienoic acids following an increase in Na^+^ concentrations in CNT plays a predominant role in the vasodilatation of Af-Art and thus increases in GFR [30,31]. Finally, whereas TGF can lead to Af-Art vasoconstriction in response to a high NaCl content in the macula densa in DCT, CTGF provokes Af-Art dilatation in response to an increase in NaCl transport in the CNT via the epithelial Na^+^ channel.

### 3.3. Renin–Angiotensin–Aldosterone System (RAAS)

The RAAS is a hormonal regulator mechanism of RBF and renal microcirculation that leads to increased sodium and water reabsorption and a subsequent increase in arterial blood pressure. RAAS also activates TGF [32]. The juxtaglomerular apparatus (JGA) consists of the macula densa (TGF) and juxtaglomerular cells (renin), which are derived from the distal tubule epithelium and smooth muscle cell of the Af-Art wall. Decreased blood pressure in the Af-Art reduces GFR and subsequently the sodium concentration in the distal convoluted tubule, which triggers the release of renin from the juxtaglomerular apparatus. The renin mediates the production of angiotensin I; angiotensin I is converted to angiotensin-II by the angiotensin-converting enzyme. The elevation of angiotensin II gives rise to a reduction in GFR by the vasoconstriction of the Af-Art and Ef-Art in the glomerulus. In addition to stimulating the antidiuretic hormone from the hypothalamus, angiotensin II is also able to stimulate the release of aldosterone from the adrenal cortex to increase sodium and water reabsorption in the collecting ducts, the loop of Henle and the distal tubules.

## 4. Sympathetic Regulation of the Renal Microcirculation

The kidney is also innervated by many nerves entering via the renal plexus [33]. The glomerular arterioles, vasa recta, macula densa cells, proximal and distal tubules and the loop of Henle are extensively innervated with sympathetic nerves [34,35]. It has been shown that the activation of renal mechanoreceptors gives rise to an increase in renal pelvic pressure and afferent neurotransmission to reduce glomerular filtration in the case of diuresis [36]. Indeed, electrical stimulation of the renal sympathetic nerves diminishes glomerular filtration and leads to water conservation [37]. Furthermore, high sodium levels can reduce the threshold for activation of the renal mechanoreceptors and thus enhance suppression of efferent sympathetic nerve to increase sodium excretion and diuresis. In contrast, a low-sodium diet causes an increased efferent sympathetic activity to sustain sodium and water retention [38].

## 5. Vascular Endothelium and Glycocalyx

**Figure 3:**
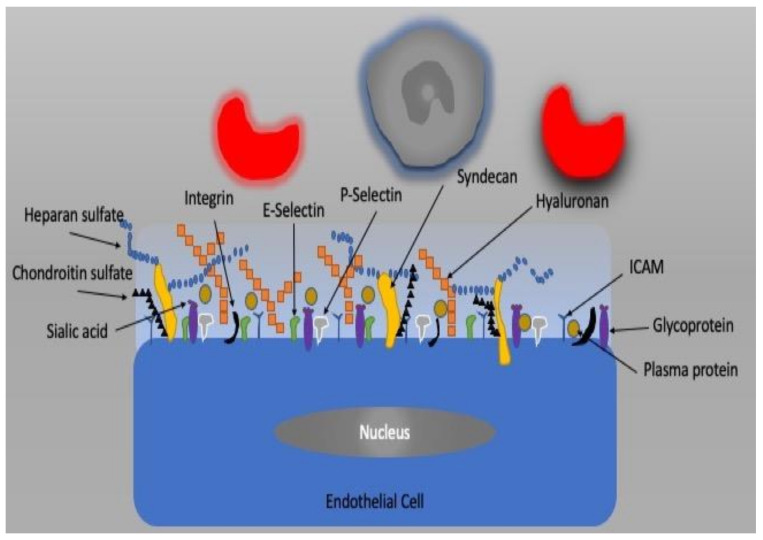
The endothelial cell and glycocalyx structure.

The vascular endothelium, a single layer of endothelial cells (ECs), is a barrier between the intra- and extravascular compartments. In addition to its barrier function, it also controls vessel diameter, transcapillary transport of macromolecules, hormones, fluid, electrolytes, and blood cells [39,40]. It is now well known that the endothelium plays an important role in the regulation of homeostasis of the microcirculation as a metabolic and endocrine organ [41]. The factors released by the endothelium change the diameter of vessels by which they modify the supply of blood to tissue. These factors include nitric oxide (NO), endothelin 1, adrenomedullin, and adenosine as well as and cyclooxygenase metabolites such as thromboxane and prostacyclin. If the balance between these mediators is disturbed, then so-called endothelial dysfunction may occur [42,43]. The apical plasma membrane of EC is covered by the glycocalyx, which is a complex network of macromolecules. It is the gel-like barrier covering the luminal side of the vascular endothelium and consists of proteoglycans, glycosaminoglycans, and plasma constituents (Figure 3). The glycocalyx creates a barrier for the transcapillary escape of water and macromolecules such as albumin and plays a role in mechanotransduction and leukocyte–endothelial interactions. Infection, ischemia/reperfusion injury, fluid overload, hemorrhage, and other vascular diseases can impair vascular endothelial barriers and cell–cell junctions as well as degrade the glycocalyx, resulting in endothelial dysfunction, which is accompanied by high vascular permeability, unresponsiveness/over-responsiveness to vasoactive mediators, endothelial cell death, leukocyte transmigration, tissue edema, and oxygen radical production, resulting in microcirculatory dysfunction, tissue hypoxia, and organ failure.

## 6. Arterial to Venous (A–V) Shunting

Arterial–venous (A–V) shunting is the mechanism that directs the transition of oxygen from the artery to the vein before the oxygenated blood reaches the microcirculatory system. A–V shunting might be seen in the cortex [44] as well as between the descending and ascending vasa recta in the medulla [45]. It plays an important role in facilitating the exchange of certain gases and other highly diffusible molecules based on their concentration gradients. Moreover, A–V shunting between the descending and ascending vasa recta appears to be critical for urinary concentrating mechanisms [46]. However, A–V shunting of oxygen (arterial-to-venous oxygen shunting) between arteries and veins in the renal cortex is thought to be an antioxidant defense mechanism that leads to the delivery of less oxygen to the microcirculatory system in case of hyperoxia, thereby reducing oxidative stress in renal tissue [47,48].

## 7. Microcirculatory Alterations during AKI

During AKI, all regulatory systems are frequently compromised due to alterations in systemic hemodynamics and renal hypoperfusion and proinflammation that leads to hypoxia, oxidative stress, endothelial and tubular damage, and, finally, the loss of function. Reduced autoregulatory capability can cause the inappropriate elevation of glomerular capillary pressure, glomerular endothelial and tubular injury, and renal dysfunction. From this perspective, the manipulation, protection, and redirection of renal autoregulatory systems may be the target of choice for both prophylaxis and treatment of renal microcirculatory dysfunction in AKI.

Microcirculatory dysfunction is characterized by heterogeneous abnormalities in renal blood supply, the alteration of renal oxygenation, capillary perfusion, flow heterogeneity, oxygen shunting, and endothelial damage. The direct effect of increased RBF on the kidney is elevated oxygen consumption and GFR. However, Lankevada et al. reported that a crucial role of intrarenal blood flow maldistribution emanating from significant hypoperfusion and hypoxia at the medullary, but not the cortical, level during AKI despite large increases in global RBF and renal oxygen delivery [49,50]. Many studies on sepsis demonstrated that RBF can be high, normal, or low according to the experimental models [50,51,52]. However, renal oxygenation is determined by oxygen delivery, consumption, and shunting [53]. The alteration of oxygenation leads to the possibility of hypoxia- or hyperoxia-mediated renal cellular damage. In particular, the outer medulla is more susceptible to damage from altered oxygen delivery due to its high metabolic requirement for maintaining efficient sodium and, particularly, water reabsorption.

Renal oxygenation is a critical factor for maintaining cell survival, renal tubular function, and glomerular filtration. However, oxygen delivery and sufficient utilization are easily compromised during the pathological or physiological events that affect the autoregulatory systems. First, RAAS activation due to volume depletion or hypotension can cause both afferent and efferent vasoconstriction leading to reduced oxygenation and perfusion of the peritubular and renal medullary regions. Second, the activation of TGF because of an increase NaCl load in distal convolute tubule due to massive fluid resuscitation or salt intake also leads to Af-Art vasoconstriction in addition to activated RAAS. Third, mechanical stimulation such as hypotension can activate both sympathetic renal nerves and myogenic regulatory mechanisms resulting in Af-Art vasoconstriction, reduced GFR, and decreased perfusion/oxygenation; in contrast, chemical stimulation based on a NaCl load causes the vasoconstriction of Ef-Art, which increases water and Na excretion. However, both can change the perfusion and oxygenation in the remaining parts of the kidney. Fourth, pathologic A–V shunting can diminish renal cortical and medullary hypoxia.

Endothelium–leukocyte interaction and activation of the coagulation system may also enhance the vasoconstriction of renal vessels leading to the local compromise of microcirculation and regional ischemia, especially in the outer medulla [54]. Furthermore, endothelial cell damage, alteration of vascular permeability, and glycocalyx degradation can also provoke AKI.

### 7.1. The Renal Microcirculation in Sepsis-Induced AKI

Septic AKI results from multiple pathological mechanisms including systemic and renal hemodynamics, intrarenal microcirculatory alterations, and renal damage. However, the changes in the intrarenal microcirculation during sepsis are not fully understood. The pathophysiology of sepsis is characterized by peripheral vasodilatation-induced hypotension, hypovolemia, tissue perfusion defect, inflammation, hypoxia, high vascular permeability-induced edema, and leukocyte transmigration [55,56]. Uncontrolled inflammation leads to increase in proinflammatory cytokines such as tumor necrosis-alpha (TNF-α) and interleukin-6 (IL-6), and it plays a critical role in sepsis pathogenesis [57,58]. AKI is a frequent complication during sepsis and is directly associated with a mortality rate of up to 40%. Recently, selective medullary ischemia and hypoxia were observed in conscious sheep with sepsis with a hyperdynamic circulation and AKI, despite an increase in total RBF [59]. In an experimental study, Heemskerk et al. showed that despite reduced renal oxygen delivery and sodium transport following bacteremia and endotoxemia, renal oxygen extraction was increased in rat kidneys [60]. Of note is that the inhibition of endothelial nitric oxide synthase (eNOS) and an increased inducible NOS synthase (iNOS), endothelial leukocyte transmigration, coagulation, glycocalyx degradation, upregulation of adhesion molecules, reactive oxygen species, and cytokines also take place in septic renal pathology [61,62,63,64].

Sepsis-induced hemorheological alterations, such as increased rigidity and aggregability of red blood cells (RBCs), can cause capillary obstructions and enhance microcirculatory derangement and shunting [65]. RBCs are also able to release NO from nitrite and thus contribute to the regulation of microvascular blood flow during a hypoxic state [66]. Fluid resuscitation and vasoactive therapies are still the cornerstone treatment of sepsis-induced impairment of systemic hemodynamics, organ perfusion, and oxygenation with normalized arterial pressure and increased intravascular volume. However, it is now well known that there is no correlation between the macro- and microcirculations, especially during sepsis [67]. Moreover, previous experiments have shown that fluid resuscitation, alone or combined with norepinephrine, is not effective for restoring renal cortical and medullary oxygenation, renal perfusion, endothelial dysfunction, or glycocalyx degradation in rats [68,69,70]. In a septic pig microcirculation study, Lima et al. first identified the presence of renal microvascular hypoperfusion and plugged vessels in the intrarenal microcirculation by means of contrast-enhanced ultrasound despite normalization of MAP and RBF [71]. The inadequacy of current treatment methods to restore renal hypoperfusion and depleted oxygen requires a focus on renal microcirculation to target new therapies such as blood transfusion, hemoglobin-based oxygen carriers (HBOCs), and antioxidant and anti-inflammatory drugs. Despite the fact that Zayed et al. did not find any significant reduction in mortality or incidence of AKI among septic patients treated with vitamin C, thiamine, and hydrocortisone, they did show a significant reduction in the SOFA score in a meta-analysis of six studies [72]. In a previous study, we demonstrated that allogenic blood transfusion was successful in correcting renal microcirculatory oxygenation and eNOS expression and prevented renal failure [73] in septic rats. Due to unwanted side effects and difficulties in the preservation of blood, HBOCs may be considered a fluid for improving both intravascular volume and organ oxygenation in perioperative and intensive care settings. In a study on septic rats, we tested the effects of PEGylated carboxyhemoglobin (PEGHbCO) on renal cortical oxygenation, muscle perfusion, and inflammation and found that it enhanced renal cortical oxygenation and restored skeletal muscle microcirculatory flow [74]. However, the biological and molecular properties of HBOCs (*p* 50 value, molecular size, oxidative capacity, and toxicity) need further optimization.

In another study, we demonstrated that the administration of the antioxidant N-acetylcysteine in addition to fluid resuscitation was able to prevent renal damage in rats by restoring renal oxygenation, tissue NO levels and hyaluronic acid (a glycocalyx degradation marker) [68]. In addition to N-acetylcysteine, recombinant alkaline phosphatase (recap) also exerted a reno-protective effect on the endotoxemic rats [75]. Recently, Lankadeva et al., demonstrated that megadose of vitamin C could improve in renal medullary PO_2_ and renal function in ovine model of sepsis [76]. To show the effects of corticosteroids on endotoxemia, we tested the effects of a bolus low-dose of dexamethasone as a supplement to fluid resuscitation in rats and found that dexamethasone improved MAP, RBF, renal cortical oxygenation, and oxygen delivery and consumption and was effective in significantly restoring renal function [77]. Recently, in a propensity-score-weighted retrospective study, we showed that a hemoadsorber (CytoSorb) filter, removed inflammatory cytokines from the blood in a continuous renal replacement therapy (CRRT) circuit, and led to a decrease in all-cause mortality at 28 days compared to CRRT alone [78]. Finally, we suggested that improved renal oxygenation and perfusion with controlled systemic and renal hemodynamics, inflammation, oxidative stress, and glycocalyx degradation, might have resulted in sufficient oxygen availability and effective oxygen utilization leading to the protection of renal integrity and function during a septic insult (Table 1).

### 7.2. Renal Microcirculation in Heart Failure and Cardiogenic Shock-Induced AKI

Heart failure-induced end-organ perfusion defects have increasingly been investigated in recent years. The pathophysiological basis of (advanced) heart failure (cardiogenic shock) includes compromised central hemodynamics and impaired organ/microvascular perfusion. Previous studies on microcirculation imaging have shown that alterations in the microcirculation have important prognostic value for patients with advanced heart failure (cardiogenic shock) as well as those receiving inotropic/mechanical circulatory support [79,80,81,82]. There is still ongoing increased morbidity and mortality alongside increasing healthcare costs following acute or chronic heart failure resulting in multi-organ failure and intensive care admissions. Artificial hearts for short- and long-term circulatory support, on top of inotropic agents (e.g., dobutamine, enoximone, milrinone, and levosimendan), have improved survival; however, the development of multi-organ failure, especially AKI, remains a point of interest influencing survival.

Short-term machinal circulatory support (MCS) devices (e.g., Extra Corporeal Membrane Oxygenation (ECMO)), as well as long-term devices (e.g., left ventricular assist devices (LVADs)), are increasingly being used as acceptable supports for acute to chronic failure and even as an alternative to heart transplantation. After stabilizing the macrocirculatory system as well as restoring microcirculation, there is often persisting multi-organ failure, particularly in AKI [83,84,85]. Innovations and treatments for (cardiogenic) shock need to be re-evaluated based on the microcirculatory responses to different components and time points of shock support. The majority of ICU admissions following cardiogenic shock are a result of myocardial infarction. Despite optimizing coronary and macrocirculatory conditions, microcirculatory defects persist and result in the need for renal replacement therapy (RRT). Recent studies have shown a significant and independent association between microcirculatory perfusion parameters (perfused capillary density, proportion of perfused capillaries, and combined clinical endpoint of all-cause death) and renal replacement therapy at 30 days follow-up [86]. In another study, Kara et al., showed that sublingual measurements of perfused vessel density can be used as predictor for ICU mortality in patients with cardiogenic shock [87]. In patients with such a loss of hemodynamic coherence, microcirculatory perfusion parameters are the most important for prognostication [86]. Therefore, there is a need for a more comprehensive approach to targeting microcirculatory perfusion defects in the kidney as a result of low capillary density, high venous congestion, high inflammation, alterations in endothelial barrier permeability, impairment of endothelium-dependent vasorelaxation, increased oxidative stress, and loss of angiogenic factors.

Following the observations of the microcirculation, there are opportunities that have to be addressed in a timely manner. Creating reperfusion by ECMO leads at the same time to high inflammation, endothelial damage, and loss of vascular response with vasoplegia, ischemia-reperfusion damage, and oxidative stress, which are also the result of cytokine storms. There is increasing evidence for the potential use of cytokine removal devices (hemoadsorbers) in this kind of shock to stabilize and prevent further organ failure [88,89,90]. In these patients, preventing further fluid resuscitation by decreasing vasoplegia will prevent venous congestion, further improve ventricular dysfunction and reduce renal injury caused by venous congestion (e.g., microcirculatory tamponade following right ventricular dysfunction).

Therefore, after decreasing vasoplegia, RRT must adequately restore fluid balance and microcirculatory venous perfusion. These combinations, used in modern intensive care, are reasonable approaches to improving survival in patients who have multiple organ dysfunction necessitating multiple organ-supportive techniques. In many studies, there were promising results for the restoration of microcirculation after cardiac arrest, decreasing post-cardiac arrest syndrome and preventing renal and intestinal damage by maintaining perfusion as well as positive inotropic and vasodilatory effects on the cardiopulmonary system and its effects on decreasing ischemic-reperfusion damage on erythrocytes [91,92,93]. To date, there is limited clinical literature and an absence of evidence from randomized trials that addresses the use of levosimendan for cardiogenic shock requiring ECMO support and weaning as well as for preventing AKI.

Furthermore, in recent years, there has been scarcity of new promising drugs for the treatment of acute heart failure and kidney injury. Serelaxin is a novel recombinant human relaxin-2 that has been investigated for the treatment of acute heart failure. However, its effects on renal function, and especially on the renal microcirculation, remain incompletely characterized. Although serelaxin increased overall RBF, urine flow, GFR, and sodium excretion, and dilated the Af-Art and Ef-Art in control conditions, these effects were attenuated or prevented in the presence of exogenous angiotensin II and NOS inhibitors [94]. We still need optimal cardiorenal monitoring to develop prevention and adequate support for acute kidney injury in patients with advanced heart failure. There is a need to understand the cellular and molecular mechanisms of renal microvascular dysfunction in acute and chronic kidney diseases during compromised cardiac conditions as well as the potential diagnostic and therapeutic implications of these findings in ICUs. Multimodal extracorporeal therapies are required for a good patient outcome after cardiogenic shock, especially when complicated by mixed features of shocks.

### 7.3. Renal Microcirculation in I/R-Induced AKI

Renal ischemia/reperfusion injury (I/R) is a common cause of AKI as a result of an imbalance in tissue oxygen supply and demand that can lead to an overproduction of reactive oxygen species and inflammatory mediators. I/R-induced AKI is associated with alterations in systemic and renal hemodynamics, disturbed renal oxygenation, inflammation, oxidative stress, impaired water and electrolyte homeostasis, and reduced excretion of metabolic waste [54,95,96,97]. Renal microvessels are also vasoconstricted in response to increased levels of endothelin-1, angiotensin II, thromboxane A2, prostaglandin H2, leukotrienes C4 and D4, and adenosine as well as sympathetic nerve stimulation [98,99,100,101].

Many studies have demonstrated the beneficial effects of different agents that can reduce inflammation and oxidative-nitrosative stress following I/R. Examples include doxycycline [102], leptin [103], levosimendan [104], iloprost [105], and ascorbic acid [106]. However, none of the studies related to these compounds focused on promoting renal oxygenation and perfusion to prevent the loss of renal function or damage during I/R-induced AKI. Therefore, in addition to inflammation and oxidative stress, we found that I/R-induced AKI in rats is associated with a low RBF, high renal vascular resistance, and diminished renal oxygenation following a supra-aortic occlusion and two hours of reperfusion [95,96,97,107,108]. Since renal oxygenation depends on a balance between oxygen supply and consumption, for which NO is a major regulator. Furthermore, Legrand et al. showed that iNOS inhibition in rats partially restored renal oxygen delivery and oxygenation and prevented deterioration of renal function [108]. The best way to reduce oxidative stress-induced AKI is to use anti-oxidant molecules that prevent the oxidation of cellular and nuclear molecules due to the overproduction of free radicals following ischemia/reperfusion. To do this, we examined the impact of TEMPOL (a superoxide scavenger) and ascorbic acid in rats on the alteration of renal microcirculatory oxygenation, damage, and loss of function in rats.

In the study, we found that the administration of TEMPOL just before the ischemia during reperfusion reduced iNOS levels, pro-inflammatory cytokines (IL-6), and myeloperoxidase (MPO) activity, thereby improving cortical and medullary oxygenation, reducing vascular resistance, and preventing renal damage [96]. Similarly, treatment with ascorbic acid led to enhanced cortical and medullary oxygenation, which correlated with reduced oxidative stress (MDA), inflammation (IL-6), MPO, and renal injury markers (e.g., neutrophil gelatinase-associated lipocalin (NGAL) and fatty acid binding protein-L (L-FABP) [97]. However, both anti-oxidants failed to improve renal oxygen delivery due to low MAP and RBF. In another study, we tested the effect of 10% hypertonic saline resuscitation on renal microcirculation and function and found that it improved MAP, RBF, microcirculatory oxygenation, renal oxygen consumption, glycocalyx integrity, and inflammation but it caused additional tubular damage in I/R-induced AKI [107].

To understand the role of inflammation, the immunosuppressant drug mycophenolate mofetil was used for prophylaxis of I/R-induced AKI in rats. We showed that oral administration before ischemia restored renal cortical oxygenation, RBF, and RVR in addition to reducing iNOS, IL-6, MPO, and renal injury markers (NGAL, L-FABP) without affecting renal function [95]. Recently, it was shown that pyridoxamine (PM), an analog of vitamin B6, diminished renal tubular damage and reduced oxidative damage after ischemia/reperfusion-induced AKI in mice [109]. In another study on rats, it was shown that vitamin D deficiency and downregulation of vitamin D receptors played a role in the occurrence of I/R-induced renal inflammation and injury [110]. By using an animal model of I/R-induced AKI, the intraperitoneal administration of low-dose doxycycline was shown to protect renal function [111]. The intracarotid administration of mesenchymal stem cells (MSCs) in a rat model of renal ischemia resulted in a significant improvement of renal function, tubular injury, and apoptosis [112]. Overall, I/R-induced AKI is associated with an imbalance of systemic and renal hemodynamics, inflammation, and oxidative stress; however, a strategy based on controlling inflammation and oxidative stress prior to the ischemic insult may prevent the occurrence of AKI in abdominal and renal surgery patients (Table 1).

### 7.4. Renal Microcirculation in Hypovolemic/Hemorrhagic Shock-Induced AKI

Hemorrhagic shock (HS), massive bleeding following trauma or major surgery, is a severe complication of AKI accompanied by high mortality and morbidity. HS is a macro- and microcirculatory failure that leads to a reduction in intravascular volume and an imbalance between cellular oxygen supply and demand. The primary treatment is a blood transfusion to normalize arterial pressure and intravascular volume and preload and to increase the oxygen-carrying capacity of the blood. However, the difficulty of finding suitable blood types and preserving and transporting fresh blood results in fluid resuscitation being the first option to increase intravascular volume, and normalize cardiac output (CO) and MAP to correct tissue perfusion and oxygenation. In the kidney, hypovolemia can cause renal perfusion defects, ischemia, and further hypoxia due to high sympathetic nerve activity and the activation of the renin–angiotensin system, which results in intrarenal vasoconstriction [113]. Recently, Wu et al. demonstrated that HS in rat kidney and intestines is accompanied by a reduction in microvascular blood flow intensity and oxygen saturation [114]. HS-induced renal cortical perfusion defect was also shown by laser speckle imaging in rats [115]. In our HS study, we investigated the effects of different types of crystalloid fluids (Ringer’s lactate, Ringer’s acetate, PlasmaLyte, and normal saline) for resuscitation after hemorrhagic shock; however, all fluids failed to improve renal cortical and medullary oxygenation despite a sufficiently improved MAP and RBF [116]. Legrand et al. also demonstrated that HS in rats is characterized by impairment of MAP, RBF, renal oxygen delivery, consumption, and microvascular PO_2_. Following resuscitation with normal and hypertonic saline to normalize the MAP at around 80 mmHg, they concluded that high-MAP-directed fluid therapy did not lead to an improvement in renal microvascular function. Furthermore, blood transfusion was unable to restore microvascular oxygenation to baseline values in an HS rat model [117]. This can be explained by persistent renal microvascular dysfunction after the hemorrhage despite normalization of the systemic hemodynamics and the oxygen-carrying capacity of blood.

Parallel to this study, we showed that renal microcirculatory alterations in rats were associated with the extent of renal damage (NGAL and tubular injury) and also inflammation (TNF-α) [117,118]. During hemorrhagic shock, a reduction in systemic and renal perfusion and oxygenation led to AKI: hypoxic tubular damage, glycocalyx degradation, and endothelial cell damage [118,119]. In our model, we demonstrated that a blood transfusion is more effective for improving microvascular oxygenation than either colloid or PEGHbCO resuscitation, but a low volume of PEGHbCO also showed positive effects on MAP, RBF, and renal damage [118]. In the end, we suggested that the additional carbon monoxide molecule, which causes vasodilation and modulates the inflammatory response, makes the PEGHbCO able to protect the kidney against hypoxic damage during the first hour of resuscitation. Additionally, Yamamoto et al. tested resuscitation with hemoglobin vesicle (HbV) that had been produced from a concentrated Hb solution covered by a lipid bilayer. They found that it improved the systemic hemodynamics, oxygen-carrying capacity of blood, oxygen delivery, and, marginally, renal cortical oxygen pressure, but it was associated with an increase in methemoglobin levels, which are toxic to the kidney [120]. In a study on rabbits, Yamamoto et al. demonstrated that despite the systemic hemodynamic achievement, HbV resuscitation showed an increase in systemic oxygen consumption and a limited positive effect on renal oxygenation with regard to the resuscitation of recombinant human albumin [121].

Another alternative for a colloidal solution is the intravenous administration of Drag-reducing polymers (DRPs), which have been shown to increase aortic and arterial blood flow and decrease peripheral vascular resistance [122]. Specifically, DRPs can improve tissue oxygenation and oxygen consumption in rats subjected to severe hemorrhagic shock [123,124,125]. Recently, Li et al. pointed out that polyethylene oxide, one of the well-known water-soluble DRPs dissolved in Ringer’s acetate, can protect the kidneys against renal injury and functional loss along with the improvement in tissue perfusion and oxygen delivery and reducing lactic acid, creatinine, TNF-α and IL-6 [126]. It was concluded that, HS-related microcirculatory derangements in the kidney required more attention to improve not only systemic hemodynamics but also renal microcirculation: perfusion, oxygenation, and endothelial function by oxygen carriers (blood or HBOCs) and anti-inflammatory and antioxidant mediators (Table 1).

### 7.5. Renal Microcirculation in Hemodilution-Induced AKI

Hemodilution is one of the main contributors to renal damage and is associated with a reduction in the oxygen-carrying capacity of blood, tissue-perfusion defects, and tissue hypoxia, resulting in compromised function. Moreover, it was shown that cardiopulmonary bypass (CPB)-related hemodilution impairs renal oxygenation and perfusion due to vasoconstriction and the redistribution of blood flow away from the kidney. The autoregulatory systems mentioned before are profoundly disturbed by acute hemodilution. In addition, the hormonal regulation of renal blood flow, composed of endocrine (systemic) as well as paracrine (locally acting) hormones, is also disturbed. During CPB-induced hemodilution, the levels of some vasoactive substances affecting renal microcirculation were altered. The level of aldosterone, which contributes to blood pressure through increasing Na^+^ and water retention, was said to be elevated during CPB [127,128]. Additionally, plasma levels of angiotensin II, which affect the efferent arteriole in particular, also increased [129]. Despite locally effective vasoactive substances such as NO [130], endothelin-1 [131], and bradykinin [132], hemodilution led to the dilution of systemically acting vasoactive substances such as angiotensin and epinephrine.

Lannemyr et al. reported that renal vasoconstriction and flow redistribution during hemodilution with Ringer’s acetate solution in CPB patients were accompanied by impairment of renal oxygen delivery, oxygen extraction, and tubular injury [133]. The effects of hemodilution on renal microcirculatory oxygenation have been studied mostly in animal models. In the first rat study, we showed that a gradual reduction in hematocrit, through replacement of blood by a colloid solution, resulted in diminished cortical and medullary oxygenation, reduced oxygen delivery, increased renal oxygen consumption, and intrarenal shunting [134]. In another study, using hemodilution with a pasteurized protein solution, we demonstrated that the kidney is much more vulnerable to a low oxygen-carrying capacity or hematocrit levels of blood compared to the intestines or heart. We found that renal oxygenation started to decrease at a Hct level of 38%, but for the heart it was 8% and for the intestines, 17% [135]. Koning et al. showed that the effects of hemodilution with or without CPB primed with a colloid solution may cause further disruption of renal microcirculation and increase inflammation, endothelial adhesion molecule expression, and renal injury in rats [136]. In another experimental study, it was shown that hemodilution with colloids was associated with low RBC aggregation, and proinflammatory and procoagulatory endothelial activation such as E–P selectin and Von Willabrand Factor (vWF) mRNA upregulation in the kidney, as well as in the heart, lungs, and liver [137]. Konrad et al. demonstrated that colloid solutions (starches) were more effective at preserving renal microvascular oxygenation and function in pigs compared to crystalloid solutions, which resulted in high tissue edema and hypoxic inducible factor expression [138]. The discrepancy between the effects of colloids and crystalloids on the renal microcirculation led us to conclude that better fluids are needed for use in perioperative and intensive care settings. During major surgery, the goal should be to avoid severe hemodilution with a reduction in the fluids used for resuscitation, using the cell salvage system, proper HBOC, or infusible oxygen-carrying fluids, to effectively achieve adequate renal perfusion and oxygenation for preventing the incidence of AKI (Table 1).

### 7.6. Renal Microcirculation in Drug-Induced AKI

Drug-induced nephrotoxicity is one of the main pathogenic causes of AKI, chronic kidney disease, and end-stage kidney disease. Nephrotoxic drugs and their metabolites are carried from the proximal tubules into distal tubules and the loop of Henle [139]. During this removal, various renal compartments such as the vascular supply, glomerulus, tubule interstitium, and collecting ducts may be negatively affected.

Renal injury is generally caused by one or more mechanisms, including alteration in glomerular hemodynamics, tubular cell toxicity, inflammation, crystal nephropathy, rhabdomyolysis, and thrombotic microangiopathy [140,141]. Aminoglycoside (AMG) antibiotics such as gentamicin cause renal tubular obstruction and vasoconstriction and mesangial contraction [142]. On the other hand, Amphotericin B as an anti-fungal drug can also induce vasoconstriction leading to decreased RBF and GFR, and causes ischemic injury [143]. The anticancer drug cisplatin may cause cell apoptosis and necrosis by activating the TNF pathway and the endoplasmic reticulum stress pathway [144].

As an example, the administration of non-steroidal anti-inflammatory drugs (NSAIDs) may impair the balance between renal vasoconstriction and vasodilation, and thus lead to renal ischemia and loss of GFR [145]. Cidofovir and adefovir both induce tubular epithelial cell apoptosis causing AKI by directly targeting proximal tubular cells [146,147]. Cyclosporine A and tacrolimus can stimulate dose-dependent acute vasoconstriction of both Af-Art and Ef-Art, leading to ischemic tubular injury and a decrease in GFR [141].

Contrast agents may increase renal tubular viscosity, thereby enhancing interstitial pressure and causing a reduction in medullary blood perfusion [148]. The higher osmolality of the contrast agents can also reduce erythrocyte deformability and increase stiffness, which results in erythrocytes not being able to pass easily through the vasa recta in the medulla. Additionally, many contrast agents can induce renal tubular cytotoxicity by medullary hypoxia, reactive oxygen species, and mitochondrial pathways [149].

Xylose–pyrogallol conjugate is an antioxidant that has recently shown potential for preventing contrast-induced AKI in rats [150]. In a meta-analysis of 10 randomized controlled trials, a prophylactic treatment with NAC in intravenous NaHCO_3_^-^ reduced contrast-induced AKI by 35% [151]. Lu et al. also showed that antithrombin III decreased the renal inflammatory response, oxidative stress, and cell apoptosis and protected RBF [152]. In addition, the administration of MSCs significantly improved renal function and reduced injury in AKI models of cisplatin [153] and glycerol [154]. The beneficial outcomes of MSC therapy on anti-inflammatory, mitogenic, anti-apoptotic, and pro-angiogenic effects was also shown in a phase 1 clinical trial (Available at http://clinicaltrials.gov/ct2/show/NCT01602328) (21 August 2021) [155].

Despite the microcirculatory effects of drug-induced AKI not being fully understood, a prevention strategy may include (1) using early kidney injury-sensitive biomarkers prior to the development of renal damage, (2) avoiding the use of high doses of drug and bolus administration, (3) alternative imaging methods without contrast agents, and (4) antioxidant or anti-inflammatory substances or MSCs that may prevent the nephrotoxicity of the drugs.

## 8. Conclusions

In our investigations, we demonstrated that hemodynamic targeted therapy alone was not sufficient for providing adequate intravascular volume, perfusion, and oxygenation to maintain renal structural integrity and function. There are many ongoing clinical trials regarding the innovative treatment of AKI in different patient populations (see the list of clinical trials in the Supplementary List S1). Microcirculatory-targeted therapies, depending on the precise nature of the condition, result in a more effective approach for preventing AKI (Table 1). Furthermore, these therapies should focus on correcting microcirculatory alterations including renal autoregulation (controlling systemic hemodynamics and angiotensin II inhibitors and reducing high NaCl), antioxidant and anti-inflammatory drugs (corticosteroids, vitamin C, vitamin E, NAC, alkaline phosphatase, mycophenolate mofetil), hemoadsorber filters, iNOS inhibition, glycocalyx protector agents, blood transfusions (taking transfusion-related reactions into account), and HBOCs. It is expected that, together with new microcirculatory diagnostic tools (e.g., hand-held vital microscopes) and early diagnostic biomarkers, novel therapeutic strategies can protect the kidney better than conventional strategies and possibly reduce the incidence of AKI.

## Figures and Tables

**Table 1:** Innovative therapies for acute kidney injury.

Types of AKI	Innovative Therapies
Sepsis-induced AKI	BTx, HBOCs, vitamins (NAC, vitamin C, and thiamine), alkaline phosphatase, corticosteroids (hydrocortisone and dexamethasone), hemoadsorption
Cardiogenic shock-induced AKI	ECMO, LVAD, hemoadsorption Serelaxin
I/R-induced AKI	iNOS inhibition (L-NIL), antioxidant agents (TEMPOL, vitamin C, pyridoxamine, and vit D), anti-inflammatory agents (MFM), iloprost, levosimendan, and leptin
Hypovolemic/hemorrhagic shock-induced AKI	HBOCs, HbV, DRPs
Hemodilution-induced AKI	HBOCs, HbV, DRPs, cell salvage
Drug-induced AKI	Antioxidant and anti-inflammatory supports, MSCs

BTx: blood transfusion; HBOCs: hemoglobin-based oxygen carriers; NAC: N-acetylcysteine; ECMO: extra-corporeal membrane oxygenation; LVAD: left ventricle assist device; I/R: ischemia/reperfusion; LNIL: L-N(6)-(1-iminoethyl)lysine hydrochloride; MFM: mycophenolate mofetil; HbV: hemoglobin vesicle; DRPs: Drag-reducing polymers; MSC: mesenchymal stem cells.

## Data Availability

Not applicable.

## Supplementary Materials

The following are available online at https://www.mdpi.com/article/10.3390/jcm10184041/s1, Supplementary List S1: The ongoing clinical trials about treatment of AKI from US National Library of Medicine, Clinicaltrial.com (accessed on 23 August 2021).
