# Supplementary material for: Kidney Microcirculation as a Target for Innovative Therapies in AKI
Source: J Clin Med. 2021 Sep 7;10(18):4041. doi: 10.3390/jcm10184041 (PMC8471583; doi:10.3390/jcm10184041)
Supplement: Supplementary file 1 [file jcm-10-04041-s001.zip › jcm-1329011-supplementary.pdf]

Supplementary List S1. The Ongoing Clinical Trials About Treatment of AKI from US National Library of Medicine, Clinicaltrial.com:

1. The Impact of Vitamin C on Postoperative Acute Kidney Injury in Risk Patients Undergoing Valvular Heart Surgery.
2. Combining CAF, L-FABP and NGAL as Potentially Diagnostic Model for Acute Kidney Injury.
3. Deferoxamine for the Prevention of Cardiac Surgery-Associated Acute Kidney Injury.
4. Study to Evaluate the Efficacy of ASP1128 (MA-0217) in Subjects at Risk for Acute Kidney Injury Following Coronary Artery Bypass Graft (CABG) and/or Valve Surgery.
5. Biochemical Effects of Remote Ischemic Pre-Conditioning on Contrast-induced Acute Kidney Injury.
6. Intravenous Administration of Vitamin B Complex Improves Renal Recovery in Patients With AKI.
7. Preop Digifab in CABG to Reduce Ouabain Levels and Prevent AKI.
8. The Role of Statins in the Prevention of Contrast-induced Acute Kidney Injury in Patients With Cardiovascular Diseases.
9. Sodium Bicarbonate for the Treatment of Severe Metabolic Acidosis With Moderate or Severe Acute Kidney Injury In ICU.
10. Acute Kidney Injury After Cardiac Surgery; A Prospective Randomized Study of Dextran Based and Crystalloid Priming Solution.
11. DyeVert System and Contrast-induced Acute Kidney Injury.
12. Erythropoietin Role in Acute Kidney Injury.
13. Acute Kidney Injury Patients Undergoing Contrast Exposure: VQ vs. CT.
14. Low Dose Vasopressin vs. Phenylephrine in Cardiac Surgery.
15. Impact of Ascorbic Acid in the Prevention of Vancomycin Induced Nephrotoxicty L-carnitine as an Adjunct Treatment for Septic Shock Patients With Acute Kidney Injury.
16. The Effects of Oxiris on Systemic Inflammation and Endothelial dysFunction.
17. CytoSorb® Reduction of FREee Hemoglobin/ Acute Kidney Injury (AKI) During Cardiac Surgery.
18. Single-Center Prospective Study to Investigate the Difference in the Incidence of Contrast-Induced Nephropathy in High-Risk Patients With the Use of the Dye-Vert Plus System.
19. (Revival) Study to Investigate the Efficacy and Safety of Alkaline Phosphatase in Patients With Sepsis-Associated AKI.
20. Q10 Preloading Before Cardiac Surgey for Kidney Failure Reduction.
21. Low Dose Albumin Versus Standard Dose Albumin in Spontaneous Bacterial Peritonitis With Acute Kidney Injury.
22. Plasmalyte Versus Saline in Trauma Patients.

23. Contrast-associated Acute Kidney Injury in Patients With Different Types of Coronary Artery Disease.
24. Elimination of Antibiotics During Renal Replacement Therapy and Cytosorb Adsorptive Therapy.
25. Safety and Efficacy of HA380 Hemoadsorption in Patients With Septic Shock.
26. Melatonin for Renal Protection in Patients Receiving Polymyxin B.
27. The Use of Inorganic Nitrate for the Prevention of Contrast-induced Nephropathy.
28. Does High-dose Vitamin B3 Supplementation Prevent Major Adverse Kidney Events During Septic Shock?
29. Effects of Red Blood Cell Transfusion on Renal Blood Flow.
30. Extracorporeal Blood Purification Therapy in Critically Ill Patients (GlobalARRT).
31. Berberine Prevent Contrast-induced Nepropathy in Patients With Diabetes.
32. Everolimus Plus Mycophenolic Acid for Kidney Preservation in Liver Transplant Recipients With Impaired Kidney Function.
33. Intravenous Amino Acid Therapy for Kidney Protection in Cardiac Surgery
